# The gut microbiome in diabetic patients with hepatocellular carcinoma: distinct bacterial compositional shifts after hepatitis C virus eradication

**DOI:** 10.3389/fmicb.2025.1693345

**Published:** 2026-01-09

**Authors:** Mohammed Ramadan, Ibrahim A. Amin, Mohamed E. Ali, Eman R. Abdelbary, Helal F. Hetta, Roba Alatawy, Fawaz E. Alanazi, Abdullah Alattar, Reem Alshaman, Yasser Alatawi, Mohammed Salah

**Affiliations:** 1 Department of Microbiology and Immunology, Faculty of Pharmacy, Al-Azhar University, Assiut, Egypt; 2 Division of Microbiology, Immunology and Biotechnology, Department of Natural Products and Alternative Medicine, Faculty of Pharmacy, University of Tabuk, Tabuk, Saudi Arabia; 3 Department of Medical Microbiology, Faculty of Medicine, University of Tabuk, Tabuk, Saudi Arabia; 4 Molecular Microbiology and Infectious Diseases Research Unit, University of Tabuk, Tabuk, Saudi Arabia; 5 Department of Pharmacology and Toxicology, Faculty of Pharmacy, University of Tabuk, Tabuk, Saudi Arabia; 6 Department of Pharmacy Practice, Faculty of Pharmacy, University of Tabuk, Tabuk, Saudi Arabia; 7 Department of Microbiology and Immunology, Faculty of Pharmacy, Port-Said University, Port-Said, Egypt

**Keywords:** diabetes mellitus, direct-acting antivirals, dysbiosis, gut microbiome, hepatocellular carcinoma

## Abstract

**Background:**

Hepatocellular carcinoma (HCC) poses a major global health burden, and diabetic patients remain at high risk even after achieving a sustained virological response (SVR) to hepatitis C virus (HCV) with direct-acting antivirals (DAAs). Gut microbiome dysbiosis is implicated in this heightened risk, but the microbial characteristics and underlying mechanisms remain poorly understood.

**Methods:**

We conducted a cross-sectional study of 81 adults divided into three groups (*n* = 27 each): (1) diabetic patients with HCC after SVR (DHCC), (2) diabetic patients without HCC after SVR, and (3) healthy controls. Stool samples and clinical data were collected approximately 3 years post-SVR. Microbial diversity, taxonomic composition, and predicted metabolic functions were analyzed and correlated with clinical markers of liver disease.

**Results:**

DHCC patients exhibited marked microbial dysbiosis, including reduced alpha diversity (Chao1, *p =* 0.003) and significant ecological disruption (Kruskal–Wallis, *p* < 0.001). The key features included enrichment of *Treponema_2* (log₂FC = 7.71, *padj* < 0.001) and *Klebsiella* (AUC = 0.88) and depletion of the butyrate-producing genus *Faecalibacterium* (2.08% vs. 9.99% in controls, *p =* 0.006). Functional analysis revealed a 56% reduction in butyrate synthesis and a 3.2-fold increase in lipopolysaccharide biosynthesis. These shifts correlated with clinical severity: *Treponema_2* abundance was associated with hepatic encephalopathy and AST levels, whereas reduced butyrate inversely correlated with FIB-4 scores. Random forest modeling identified *Klebsiella*, *Enterobacter*, and *Megasphaera* as the top predictors of DHCC.

**Conclusion:**

Diabetic patients with HCC after HCV eradication exhibit a persistent gut microbiome signature characterized by proinflammatory and carcinogenic features. These findings highlight potential targets for microbiome-based risk stratification and therapeutic interventions in this high-risk population.

## 1 Introduction

Hepatocellular carcinoma (HCC) is a significant global health concern, ranking as the fourth leading cause of cancer-related deaths worldwide (Liu et al., 2023). The increasing incidence of HCC among patients with metabolic dysfunction, especially those with diabetes mellitus, presents a critical clinical challenge. Diabetic individuals have been shown to have a 2- to 3-fold greater risk of developing HCC than nondiabetic individuals do (Mohammed et al., 2018; Zang et al., 2023). This risk is further amplified in the context of chronic hepatitis C virus (HCV) infection, where the combination of viral hepatotoxicity and diabetes-associated metabolic disturbances creates a permissive environment for hepatocarcinogenesis (Ali and Mohamed, 2019; Thrift et al., 2023). The interplay between diabetes and HCV in shaping HCC risk after DAA-induced SVR remains unclear, underscoring the need to identify microbiome-based biomarkers for improved surveillance in this vulnerable population.

Chronic HCV infection affects approximately 58 million individuals globally and remains a major contributor to cirrhosis and HCC (Blach et al., 2022). DAAs have revolutionized the treatment of HCV, achieving SVR rates exceeding 95%. However, emerging evidence indicates that metabolic comorbidities, such as diabetes, may reduce the protective effects of achieving viral clearance on the risk of HCC (Medrano et al., 2021). Additionally, HCV-induced metabolic changes, including insulin resistance and hepatic steatosis, may continue in many patients after they achieve SVR, particularly in those with preexisting diabetes (Kralj et al., 2016). These observations highlight the need to identify novel biomarkers that can stratify HCC risk in diabetic patients following HCV eradication, as current surveillance strategies based solely on fibrosis may be inadequate for this growing patient population.

The gut microbiome plays a crucial role in regulating metabolic and liver health. Increasing evidence connects microbial imbalances, or dysbiosis, to the progression of diabetes and the severity of liver disease (Aron-Wisnewsky et al., 2020; Wu et al., 2021). In individuals with diabetes, the gut microbiome typically exhibits lower microbial diversity, a decrease in the producers of short-chain fatty acids (SCFAs), and an increase in harmful organisms, known as pathobionts, which can compromise intestinal barrier function (Allin et al., 2015; Elsherbiny et al., 2025). These alterations may be further exacerbated by HCV infection, which has been shown to independently perturb gut microbial communities through mechanisms involving bile acid metabolism and systemic inflammation (Aron-Wisnewsky et al., 2020). The resulting gut–liver axis dysfunction may represent a shared pathway through which diabetes and HCV infection synergistically promote hepatocarcinogenesis, although this hypothesis requires rigorous investigation in well-characterized clinical cohorts.

The gut–liver axis facilitates bidirectional communication between the intestinal microbiota and liver function, with microbial metabolites affecting liver inflammation, fibrosis development, and cancer signaling (Albillos et al., 2020; Martín-Mateos and Albillos, 2021). In HCV-infected individuals, gut barrier impairment and microbial translocation have been associated with more rapid fibrosis progression and impaired antiviral immune responses (Bajaj et al., 2021). Diabetes compounds these effects through glucose-mediated shifts in microbial ecology and increased intestinal permeability, creating a proinflammatory milieu that may persist after HCV eradication (Zhou et al., 2020). Recent work has identified specific microbial signatures associated with HCC development in cirrhotic patients (Ren et al., 2019; Kang et al., 2022), but whether these signatures differ between diabetic and nondiabetic populations or are modified by DAA therapy remains unknown.

The current understanding of microbiome–HCC interactions in diabetic patients is limited by several gaps in knowledge. Most studies have focused on nondiabetic cohorts or failed to account for virological status, whereas those examining post-DAA microbial changes have not specifically addressed diabetes-related modifications (Kanwal et al., 2017; Ponziani et al., 2019). Furthermore, existing microbiome-based HCC prediction models do not incorporate metabolic parameters that may significantly influence microbial risk signatures in diabetic populations (Liu et al., 2023). This knowledge gap is particularly concerning, given the increasing prevalence of metabolic dysfunction among HCV patients and the recognition that diabetes may accelerate liver disease progression even after successful viral clearance (Yang et al., 2024).

This study aimed to investigate the gut microbiome composition and functional potential of DHCCs who achieved SVR following DAA therapy. These patients were compared with DAA-treated diabetic individuals without HCC and healthy controls to elucidate how diabetes and prior HCV infection contribute to persistent microbial dysbiosis and influence HCC risk. The ultimate goal was to better understand gut–liver axis alterations in this high-risk population and explore their implications for future risk stratification and prevention strategies.

## 2 Materials and methods

### 2.1 Study design and participant recruitment

This was a cross-sectional study of patients who achieved a sustained virological response (SVR) following direct-acting antiviral (DAA) therapy and subsequently developed hepatocellular carcinoma (HCC). All the data and stool samples were collected at a single time point, approximately 3 years after SVR, to capture long-term microbial patterns rather than transient posttreatment changes. The study included 81 adults divided into three groups (*n* = 27 each): (i) DHCCs, diabetic patients with HCC after SVR; (ii) diabetic controls, diabetic patients without HCC who also achieved SVR; and (iii) healthy controls without diabetes, HCC, or hepatitis C virus (HCV) infection. Controls were matched by age, sex, and body mass index (BMI) to minimize demographic and metabolic confounding. All healthy controls were confirmed to have no history of diabetes (HbA1c < 6.5%), HCV infection, liver disease, or gastrointestinal disorders. Participant evaluations and stool sample collection occurred during the enrollment period (January 2021–December 2024). For the DAA-treated groups, SVR was defined as an undetectable level of HCV RNA (<15 IU/mL) at 12 weeks post-therapy, which was verified from medical records. The median time from SVR to sampling was approximately 3 years. All HCC cases in the DHCC group were prevalent and were diagnosed before enrollment. The sample size was estimated *a priori* using a power analysis for microbiome studies performed with the shinyMB application^1^ (Mattiello et al., 2016). The analysis was configured for a case–control study of stool microbiomes, testing for differences in the most abundant taxa with an effect size of 20–40% relative abundance change. This simulation indicated that with 25–40 samples per group, the study would be powered to detect these community-level changes (Kelly et al., 2015). Participants were recruited from the Internal Medicine Department, Mabarah Insurance Hospital, Zagazig, Egypt, between January 2021 and December 2024. Participants were eligible for inclusion if they were adults between 30 and 75 years of age. For the two patient groups, a confirmed diagnosis of type 2 diabetes mellitus, defined by an HbA1c level of 6.5% or higher, was established. Specifically, for the DHCC group, inclusion was contingent upon a diagnosis of hepatocellular carcinoma confirmed either histologically or radiologically, alongside documented SVR. Additionally, the inclusion criteria required documented HCV RNA status before and after DAA therapy and complete clinical records documenting the regimen (400 mg sofosbuvir plus 60 mg daclatasvir, with or without ribavirin) per Egyptian national guidelines (EASL, 2017; Abdel-Moneim et al., 2018). For the diabetic group without HCC, individuals must have achieved SVR and have no radiological or clinical evidence of liver malignancy. A final requirement for all participants was the absence of any antibiotic, probiotic, or prebiotic use for a minimum of 3 months before enrollment and sample collection. Dietary intake was assessed using standardized food frequency questionnaires at baseline and follow-up to ensure adherence to the Mediterranean diet (Formisano et al., 2024).

All potential participants were excluded on the basis of several factors to control for confounding variables. These exclusions included active coinfection with hepatitis B virus (surface antigen positive) or HIV; the presence of other liver diseases, such as autoimmune hepatitis, primary biliary cholangitis, Wilson’s disease, or alpha-1-antitrypsin deficiency; and a history of chronic gastrointestinal disorders, including inflammatory bowel disease, celiac disease, or prior major intestinal resection. Furthermore, individuals using immunosuppressive medications were also excluded from the study. Furthermore, given the known impact of obesity on the gut microbiota, individuals with a body mass index (BMI) > 30 kg/m^2^ were excluded. Although alcohol consumption is culturally low in the study population, self-reported alcohol use was not documented. Participants with significant ongoing alcohol consumption (defined as >30 g/day for men and >20 g/day for women) or a history of alcohol-related liver disease were excluded from the study to minimize this confounder and to ensure a homogeneous cohort. All participants provided written informed consent, and the study protocol was approved by the ethics committee at the Faculty of Pharmacy, Port Said University (Reference no. F-9-2020), under the Declaration of Helsinki ethical principles for medical research (World Medical, 2013).

### 2.2 Clinical and biochemical assessments

A comprehensive clinical evaluation was performed on all participants, including liver imaging studies (ultrasound, CT, or MRI), transient elastography (FibroScan), and standard laboratory testing. Blood samples were collected after an overnight fast for measurement of liver enzymes (ALT and AST), synthetic function markers (albumin, bilirubin, and INR), metabolic parameters (HbA1c and fasting glucose), and complete blood counts. The FIB-4 index was calculated for all participants using a standard formula that incorporates age, AST, ALT, and platelet values. HCC diagnosis was confirmed either histologically or radiologically according to international standards and Egyptian clinical practice guidelines (Akel et al., 2018). For patients in the DHCC group, disease severity was further characterized using the Child–Pugh classification and Barcelona Clinic Liver Cancer (BCLC) staging system (Yamakado et al., 2014). The majority of DHCCs had early-stage HCC (BCLC stage A) and compensated liver disease (Child–Pugh A), creating a relatively homogeneous cohort for microbiome analysis. All laboratory assays were performed in certified clinical laboratories using standardized methodologies (Kotlyarov, 2024; Wang et al., 2025).

### 2.3 Stool sample collection and DNA extraction

Fecal samples were collected from participants using sterile DNA/RNA Shield collection kits (Zymo Research, Irvine, CA, USA) following the manufacturer’s instructions. The samples were immediately placed in insulated containers with ice packs and transported to the central processing laboratory within 2 h of collection. Upon receipt, samples were aliquoted and stored at −80 °C until DNA extraction. Microbial DNA was extracted from 200 mg of fecal material using the QIAamp PowerFecal Pro DNA Kit (Qiagen, Hilden, Germany) according to the manufacturer’s protocol, including a mechanical lysis step with bead beating for 5 min at 30 Hz. DNA concentration and purity were assessed using both NanoDrop spectrophotometry (Thermo Fisher Scientific, Waltham, MA, USA) and Qubit fluorometry (Thermo Fisher Scientific), with all samples demonstrating A260/280 ratios between 1.8–2.0 and minimum concentrations of 10 ng/μL. To monitor contamination, negative blanks for DNA and PCR were included in each processing batch.

### 2.4 16S rRNA gene sequencing and data processing

The V3-V4 hypervariable regions of bacterial 16S rRNA genes were amplified using the forward primer 5′ TCGTCGGCAGCGTCAGATGTGTATAAGAGACAGCCTACGGGNGGCWGCAG and the reverse primer 5GTCTCGTGGGCTCGGAGATGTGTATAAGAGACAGGACTACHVGGGTATCTAATCC (the underlined bp indicates Illumina adapter sequences) (Ramadan et al., 2019). PCR amplification was performed in triplicate in 25 μL reactions containing 12.5 ng template DNA, with cycling conditions consisting of initial denaturation at 95 °C for 3 min, followed by 35 cycles of denaturation at 95 °C for 30 s, annealing at 55 °C for 30 s, and extension at 72 °C for 30 s, with a final extension at 72 °C for 5 min. The amplification products were visualized on 1.5% agarose gels, pooled in equimolar concentrations, and purified using AMPure XP beads (Beckman Coulter, Brea, CA, USA)(Ramadan et al., 2016). Sequencing was performed on the Illumina MiSeq platform (Illumina, San Diego, CA, USA) at IGA Technology Services (Udine, Italy).

### 2.5 Bioinformatics analysis

Raw sequencing data were processed using the QIIME2 pipeline (version 2022.2). Sequence reads were demultiplexed using the q2-demux plugin, followed by quality filtering, denoising, and chimera removal using DADA2 with default parameters (Callahan et al., 2016; Ramadan et al., 2019). The resulting amplicon sequence variants (ASVs) were aligned and assigned taxonomy using a naive Bayes classifier trained on the SILVA 138.1 reference database with a 97% sequence similarity group (Quast et al., 2013). Alpha diversity (Observed ASVs, the Shannon diversity index, and the Chao1 estimator), richness estimator, and *β*-diversity were applied at a standardized sequencing depth of 27,028 reads per sample to preserve diversity metrics using the phyloseq package (v1.36.0) (Supplementary Figure S1) using the q2-diversity plugin (Callahan et al., 2016). Beta diversity differences between groups were assessed using Permutational Multivariate Analysis of Variance (PERMANOVA) as implemented in the adonis2 function (vegan package). To ensure that the observed microbial separation was not driven by other factors, the model was adjusted for the following covariates: age (continuous), sex (categorical), body mass index (BMI) (continuous), and the use of key medications (metformin, insulin, statins, and PPIs; all coded as categorical yes/no variables). The analysis was based on Bray–Curtis dissimilarities and used 999 permutations. The significance of the disease status was tested after accounting for all other variables in the model (using the “margin” option) (Anderson, 2001). To determine whether the observed differences were driven by group dispersion (homogeneity of variances) in addition to location, Permutational Analysis of Multivariate Dispersions (PERMDISP) was also performed.

The functional potential of microbiomes was inferred by applying Tax4Fun (Aßhauer et al., 2015) with the Kyoto Encyclopedia of Genes and Genomes (KEGG) pathway (Kanehisa and Goto, 2000). Enterotyping was conducted using PAM clustering of Jensen–Shannon divergence distances from genus-level relative abundances, with the optimal cluster number determined by the silhouette width and Calinski–Harabasz index (Arumugam et al., 2011).

### 2.6 Machine learning classification analysis

Two complementary machine learning approaches were employed to increase robustness and reproducibility. First, MicrobiomeAnalyst’s integrated pipeline (scikit-learn implementation) was used for initial feature selection and classification (500 trees, Gini impurity) (Chong et al., 2020). Second, a custom random forest model was implemented in R using the randomForest package to validate findings and assess feature importance (Liaw and Wiener, 2002). Both approaches were applied at the genus level. Preprocessing includes filtering nonvariable features and addressing class imbalance through class weighting, stratified data splits, and balanced subsampling (Liu et al., 2022; Akinwumi et al., 2025). For data preprocessing and class imbalance handling, the genus-level abundance table was filtered to remove nonvariable features. To address potential class imbalance and mitigate overfitting, we employed a comprehensive strategy: (1) class weights were applied inversely proportional to class frequencies; (2) all data splits were stratified by outcome; and (3) balanced subsampling was used during model training, with each tree built using an equal number of observations from each class (Liu et al., 2022; Akinwumi et al., 2025). Model training and validation were performed through stratified 5-fold cross-validation repeated 10 times (50 total iterations). Additionally, an independent hold-out validation was performed using a 70/30 stratified split. Model performance was evaluated using the area under the receiver operating characteristic curve (AUC-ROC), with 95% confidence intervals (CIs) calculated using DeLong’s method. All random processes were executed with a fixed random seed (123) to ensure reproducibility. Permutation testing (1,000 iterations) was performed to establish chance-level AUC distributions and assess statistical significance (Singh et al., 2021). Feature importance was determined by the mean decrease in accuracy, which was averaged across cross-validation folds (Supplementary Table S1, Supplementary Figure S2). *Post hoc* false discovery rate (FDR) correction was applied to account for multiple comparisons in the feature importance analysis.

### 2.7 Statistical analysis

All the statistical analyses were performed in R (version 4.2.2) (McMurdie and Holmes, 2013). Continuous variables were compared between groups using the Kruskal–Wallis test with Dunn’s *post hoc* correction for multiple comparisons. Categorical variables (male sex) are presented as counts (percentages) and were analyzed using the chi-square test. Differential abundance testing of microbial taxa was conducted on nonrarefied counts using DESeq2 with negative binomial Wald tests and Benjamini–Hochberg false discovery rate correction (Benjamini and Hochberg, 1995; Love et al., 2014). Similarly, linear discriminant analysis effect size (LEfSe) was applied at the genus level (LDA score cutoff ≥ 2.0, *p <* 0.05) to define potential microbial biomarkers (Segata et al., 2011). Spearman’s rank correlation was used to assess relationships between microbial features and clinical parameters (*p <* 0.05). Differential abundance analysis was performed using MaAsLin2 (Microbiome Multivariable Association with Linear Models) in R (Mallick et al., 2021). The model included disease status as the primary fixed effect, with age, sex, BMI, and key medications (metformin, insulin, statins, and PPIs) included as additional fixed effects to control for potential confounding factors. Data normalization was applied using Total Sum Scaling (TSS), followed by log transformation. Associations were tested using linear models, and multiple testing correction was applied using the Benjamini–Hochberg method. The results were filtered on the basis of q values (< 0.05) for significance. To investigate the associations between the gut microbiota and clinical parameters, two complementary approaches were applied. First, a correlation heatmap was generated using the top 25 genera ranked by mean relative abundance across all samples. Spearman correlation coefficients were calculated between genus-level relative abundances and demographic, clinical, and disease status variables. Correlation matrices were visualized using the pheatmap package in R with a diverging color scale (blue = negative, red = positive, white = near zero; range: −1– +1).

Second, genus-level correlation networks were constructed separately for the DHCC and diabetic groups to explore co-occurrence patterns. For each group, the dominant genera were selected on the basis of mean relative abundance (the top 25 genera were ranked by mean relative abundance across all samples). Spearman correlations were computed between genera, and only edges with q ≥ 0.6 were retained. The networks were visualized using the igraph package in (Gajdo et al., 2015; Mallick et al., 2021). *p* values are presented throughout the text, with values below 0.001 reported as *p* < 0.001.

### 2.8 Availability of data and materials

The 16S rRNA raw sequences have been deposited in the NCBI BioProject database (https://www.ncbi.nlm.nih.gov/bioproject/PRJNA1305475). The analysis scripts are available at https://gist.github.com/Mohammedramadan2012.

## 3 Results

### 3.1 Clinical and biochemical characteristics across study groups

The clinical and biochemical characteristics of the study cohort are summarized in Table 1. As expected, the markers of liver disease significantly varied. Compared with that in the controls, the serum AST level was elevated in both patient groups (Kruskal–Wallis test, *p =* 0.0038), with the highest level found in the DHCC group. A progressive increase in total bilirubin from the control group to the DHCC group was also observed (*p* < 0.001). The DHCC group presented the most pronounced signs of advanced hepatic impairment, characterized by significantly lower platelet counts (*p* < 0.001), hypoalbuminemia (*p* < 0.001), and an elevated international normalized ratio (INR) (*p* < 0.001). Consequently, the noninvasive fibrosis index FIB-4 was significantly highest in the DHCC group (*p =* 0.0052).

**Table 1:** Clinical and biochemical characteristics by study group.

Parameter	Control (*n* = 27)	Diabetic (*n* = 27)	DHCC (*n* = 27)	*p*-value
Age (years)	41.3 ± 10.8	40.1 ± 11.2	45.8 ± 14.6	0.423
Male sex	13 (48.1%)	12 (44.4%)	14 (51.9%)	0.512
BMI (kg/m^2^)	22.5 ± 1.8	26.8 ± 2.1	27.1 ± 2.4	<0.001
HbA1c (%)	5.2 ± 0.4	7.8 ± 1.2	7.9 ± 1.5	<0.001
Platelets (×10^9^/L)	250 ± 30	218 ± 47	165.8 ± 66.3	<0.001
AST (IU/L)	25 ± 5	42.6 ± 24.7	59.6 ± 32.9	0.0038
ALT (IU/L)	22 ± 5	38 ± 20	55 ± 30	0.005
Albumin (g/dL)	4.5 ± 0.3	4.1 ± 0.4	3.9 ± 1.1	<0.001
Total Bilirubin (mg/dL)	0.7 ± 0.1	1.2 ± 0.3	1.55 ± 0.5	<0.001
FIB-4	1.29 ± 0.2	3.6 ± 2.5	5.08 ± 1.9	0.0052
INR	1.0 ± 0.1	0.98 ± 0.1	1.18 ± 0.2	<0.001

Continuous variables (Age, HbA1c, Platelets, AST, ALT, Albumin, Total Bilirubin, FIB-4, INR) are presented as the means ± SDs and were analyzed using the Kruskal–Wallis test. Categorical variables (male sex) are presented as counts (percentages) and were analyzed using the chi-square test.

#### 3.1.1 Gut microbiome dynamics in diabetic patients with and without HCC

**Figure 1:**
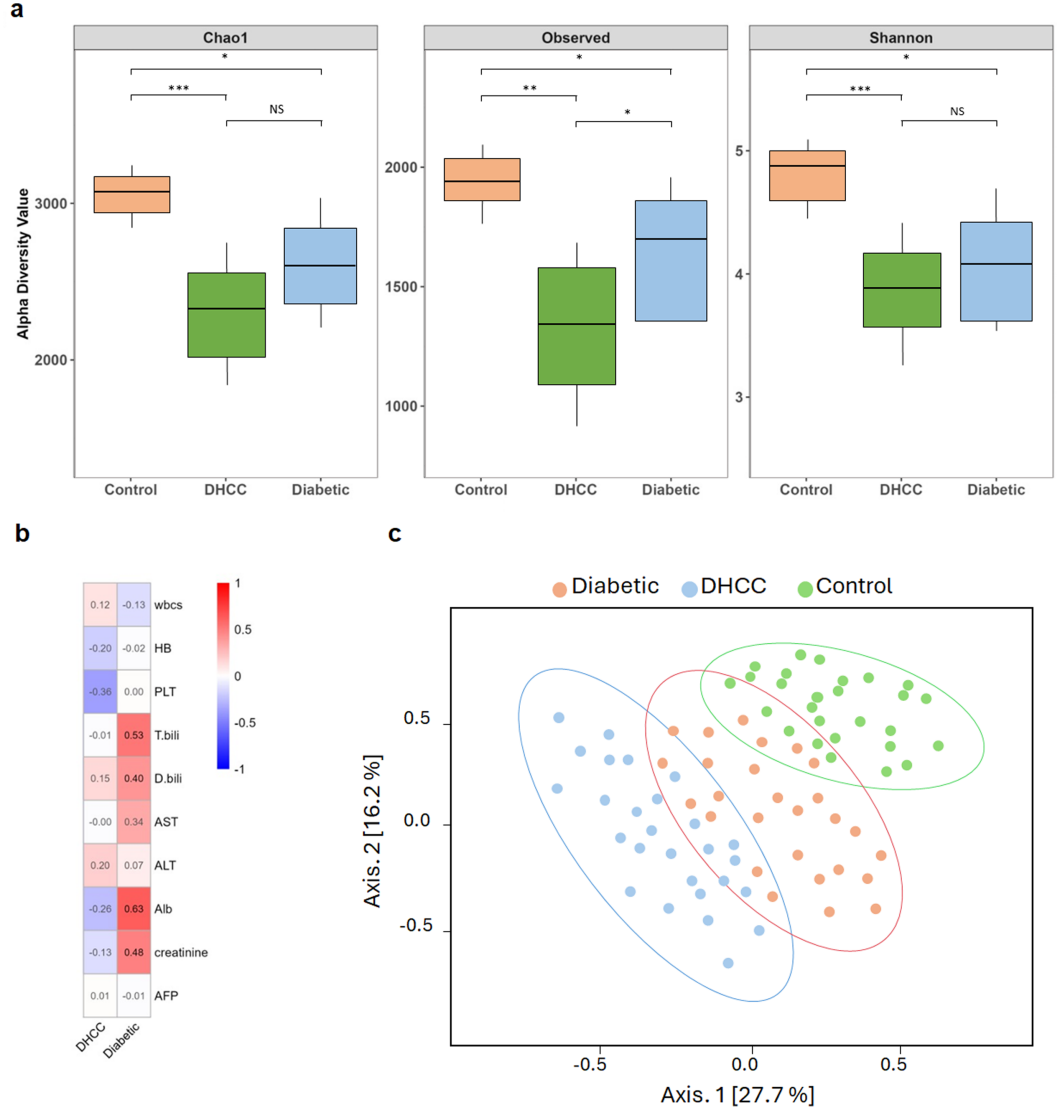
Microbiome diversity and clinical associations with HCC progression. **(a)** Boxplots display the alpha diversity indices, namely, the Shannon, Chao1, and observed species indices, across three cohorts: healthy controls, DHCCs, and diabetic patients. Each box represents the interquartile range (IQR), with whiskers extending to 1.5 times the IQR. **(b)** Heatmap show the Spearman correlation coefficients between the Shannon diversity indices of individual samples and key clinical variable groups. The color gradient indicates the direction and strength of the correlations, with red representing positive correlations and blue representing negative correlations. **(c)** Principal coordinate analysis (PCoA) plots of the beta diversity of the gut microbiota, with 95% confidence ellipses, show group-associated clustering. PERMANOVA confirmed significant compositional variation (*F* = 2.099, *R*^2^ = 0.176, *p =* 0.00184), whereas PERMDISP indicated significant differences in group dispersion (*p =* 0.00263), reflecting ecological instability in HCC patients. The first two principal coordinates account for 27.7 and 16.2% of the total variance, respectively.

The gut microbiome exhibited significant ecological shifts across diabetic status groups. Healthy controls maintained the highest microbial diversity (Shannon index: 4.71 ± 0.38; Chao1 richness: 2940.61 ± 176.12), whereas diabetic patients presented marginally reduced diversity (Shannon index: 4.32 ± 0.50; Chao1 index: 2422.57 ± 409.87) (Figure 1a). In contrast, DHCC displayed markedly reduced microbial diversity (Chao1: 2158.35 ± 568.03 vs. controls, *p =* 0.003) and greater variability in Shannon diversity (Wilcoxon rank sum test, *p =* 0.08). The Chao1 estimator effectively distinguished DHCCs from controls (AUC = 0.79, 95% CI: 0.65–0.93).

#### 3.1.2 Gut microbiome signatures reflect diabetic liver disease severity

Microbial richness correlated with clinical markers of metabolic dysfunction. To visualize the relationships between alpha diversity and clinical parameters, a heatmap was generated to display the Spearman correlation coefficients between the Shannon diversity index of each sample and key clinical variables across all participants (Figure 1b). DHCC presented a 26% reduction in ASV richness compared with controls (Chao1: 2158.35 ± 568.03 vs. 2940.61 ± 176.12, *p =* 0.003), in parallel with advanced liver disease severity. Notably, diabetic patients without HCC presented intermediate richness (Chao1: 2422.57 ± 409.87), with inverse correlations with metabolic markers (Chao1 index and HbA1c levels within the diabetic non-HCC group*; r =* −0.54, *p =* 0.02). Shannon diversity overlapped between groups, but DHCCs presented significantly lower observed species counts (Kruskal–Wallis test, *p* < 0.001).

#### 3.1.3 Microbial community architecture predicts disease subtypes

Beta diversity analysis clearly revealed a separation in the microbial community structure according to diabetic/HCC status (PERMANOVA: *F* = 2.099, *R*^2^ = 0.176, *p =* 0.00184) (Figure 1c). This significant PERMANOVA result was corroborated by a significant PERMDISP test (*F* = 1.822, *p =* 0.00263), indicating that the groups differed not only in their central microbial composition but also in their internal dispersion. Together, these findings potentially provide evidence that the observed beta diversity differences are driven primarily by disease state rather than confounding factors such as medication regimens or metabolic parameters.

#### 3.1.4 Alterations in the gut microbiome in DHCCs

**Figure 2:**
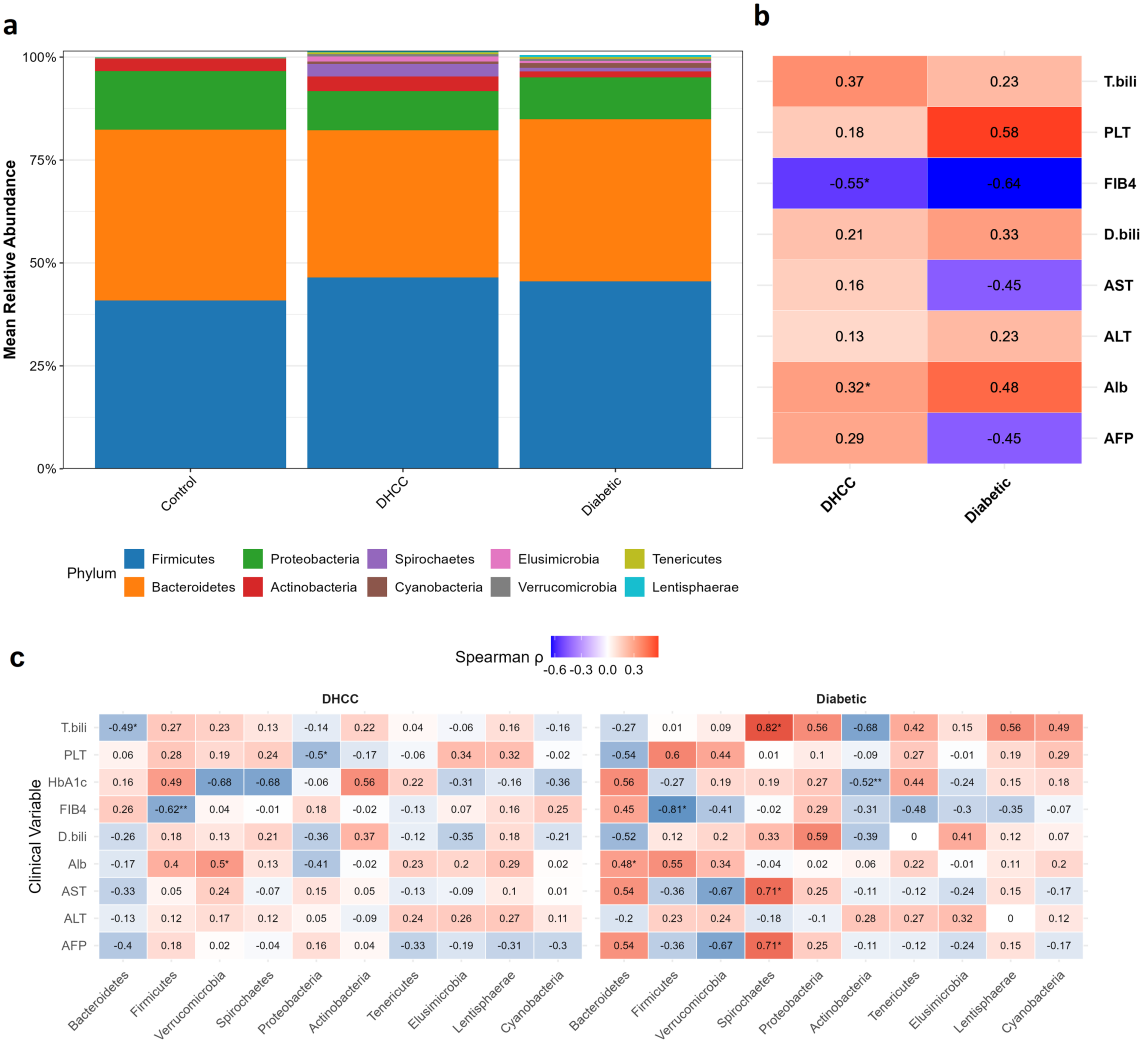
Gut microbiota composition and its clinical correlation with HCC progression. **(a)** Stacked bar plots illustrate the relative abundance of the seven most dominant bacterial phyla across three clinical groups: healthy controls, diabetic patients, and diabetic patients with hepatocellular carcinoma (DHCC). **(b)** Spearman correlation heatmap demonstrates the relationships between the Firmicutes-to-Bacteroidetes (F/B) ratio and key clinical biomarkers, including platelet count (PLT), total bilirubin (T. bili), aspartate aminotransferase (AST), alanine aminotransferase (ALT), albumin (Alb), creatinine, and alpha-fetoprotein (AFP). The correlation coefficients (r) are annotated, with statistical significance indicated by asterisks (*p < 0.05, **p < 0.01). **(c)** Heatmap show Spearman correlation coefficients (*ρ*) between the relative abundances of the top 10 bacterial phyla and selected clinical variables across study groups. The clinical variables included alpha-fetoprotein (AFP), albumin (Alb), alanine aminotransferase (ALT), aspartate aminotransferase (AST), direct bilirubin (D. bili), the fibrosis-4 index (FIB4), the platelet count (PLT), total bilirubin (T. bili), and hemoglobin A1c (HbA1c). Each cell represents the correlation value, with color intensity indicating the strength and direction of the association (blue = negative, red = positive). Significance levels are denoted by asterisks (*p < 0.05, **p < 0.01, ***p < 0.001). Groups are stratified by diabetic status (diabetic, DHCC).

The sequencing of the stool samples generated 2,760,561 reads (mean ± SD; 34,081 ± 7,417). Quality filtering and removal of ambiguous and chimeric sequences resulted in 2,374,082 reads. Taxonomic assignment of high-quality reads revealed 21 phyla, 37 classes, 75 orders, 174 families, 538 genera, and 1,696 unique ASVs. The gut microbiome exhibited distinct compositional shifts across diabetic and HCC disease states (Figure 2a). Compared with healthy controls, the DHCC group presented a significantly lower abundance of Bacteroidetes (35.7 ± 12.6% vs. 41.5 ± 14.1% in controls; Kruskal–Wallis test, *p =* 0.021). Notably, DHCC presented an enriched abundance of Spirochaetes (Kruskal–Wallis, *p* < 0.001) and Elusimicrobia (1.2 ± 2.1%), which were substantially absent in the control group.

#### 3.1.5 Clinical correlations and disease-associated microbial signatures

The Firmicutes/Bacteroidetes (F/B) ratio increased progressively from controls (0.98) to diabetic patients without HCC (1.15) to HHCCs (1.30, *p =* 0.006) (Figure 2b). Interestingly, in DHCCs, the F/B ratio was negatively correlated with the FIB-4 score (*r =* −0.55, *p =* 0.025) and a positive correlation with the serum ALB concentration (*r =* 0.32, *p =* 0.021). On the basis of the comprehensive correlation analysis between phylum-level abundances and clinical parameters, several significant associations were identified (Figure 2c). In the DHCC group, Bacteroidetes was significantly negatively correlated with total bilirubin (*r =* −0.487, *p =* 0.047), whereas Firmicutes was strongly negatively correlated with FIB-4 score (*r =* −0.625, *p =* 0.009). Additionally, Proteobacteria abundance was negatively associated with platelet count (*r =* −0.498, *p =* 0.042), and Verrucomicrobia abundance was positively correlated with the ALB level (*r =* 0.504, *p =* 0.039). In the diabetic non-HCC group, Actinobacteria exhibited a significant inverse relationship with HbA1c (*r =* −0.52, *p =* 0.008), and Spirochaetes demonstrated strong positive correlations with both AFP (*r =* 0.710, *p =* 0.048) and total bilirubin (*r =* 0.821, *p =* 0.012). These findings reveal distinct phylum-associated clinical associations that differentiate the microbial ecology of diabetic patients with and without HCC, highlighting potential mechanistic links between the gut microbiota composition and disease progression.

### 3.2 Genus-level microbiome alterations in DHCCs

**Figure 3:**
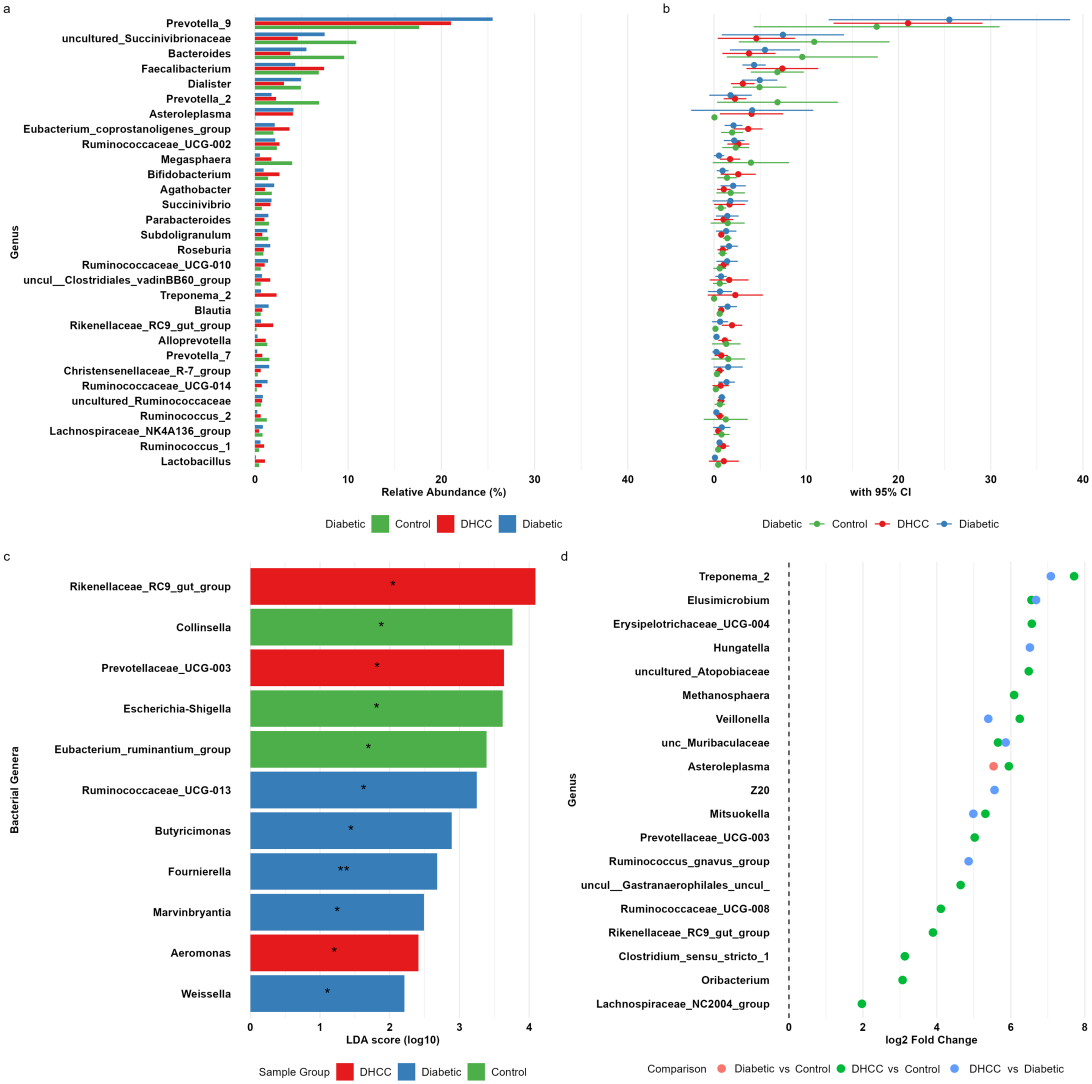
Comparison and differential abundance of the gut microbiome across clinical groups. A multipanel plot illustrates the differential abundance of gut bacterial genera across the studied groups. **(a)** Bar plot depicts the mean relative abundance (%) of the dominant genera across sample groups, with asterisks denoting statistical significance. **(b)** Confidence interval plot shows the 95% CI for genus-level abundance, highlighting variability across groups. **(c)** LEfSe bar plot presents genera with significant LDA scores (log10), indicating potential biomarkers enriched in each study group, with asterisks denoting statistical significance (*p < 0.05, **p < 0.01). **(d)** Dot plot (volcano-style) represents the significantly differentially abundant genera identified using DESeq2, which is based on log2-fold changes between groups.

Genus-level analysis revealed greater taxonomic disturbances in DHCCs than in diabetic non-HCC patients and control individuals (Kruskal–Wallis test: *p* < 0.001) (Figures 3a,b). *Prevotella_9* was dominant in all cohorts but showed significant abundance variation (Kruskal–Wallis; *p =* 0.004), with the highest levels in diabetic patients (25.51% ± 6.65) versus DHCCs (21.03% ± 4.09) and controls (17.63% ± 6.77). While the core commensals *Roseburia* and *Subdoligranulum* remained stable, the butyrate-producing *Faecalibacterium* was markedly depleted in DHCCs (2.08% vs. 9.99% in controls; DESeq2, log₂FC = −3.2, *padj* = 0.006), corresponding to a 9.2-fold lower abundance.

LEfSe confirmed DHCC-associated enrichment of *Rikenellaceae_RC9_gut_*group (LDA = 4.11, *p =* 0.039) and *Prevotellaceae_UCG_003* (LDA = 3.71, *p =* 0.024; DESeq2 *padj* = 0.017), with concomitant depletion of *Collinsella* (LDA = 3.80, *p =* 0.015) (Figure 3c). For diagnostic modeling, random forest analysis identified *Klebsiella* (AUC = 0.88), *Enterobacter* (AUC = 0.85), and *Megasphaera* (AUC = 0.82) as the top predictors, whereas *Dorea* (AUC = 0.76, *p =* 0.037), *Prevotellaceae_UCG_003* (AUC = 0.77, *p =* 0.041), and *Ruminococcaceae_UCG_014* (AUC = 0.76, *p =* 0.044) showed significant discriminatory power (Supplementary Figure S3).

The most striking genus-level enrichment was observed for *Treponema_2*, whose abundance was 209-fold greater in DHCCs than in healthy controls (Figure 3d) (DESeq2, log₂FC = 7.71, *padj* <0.001). When DHCCs were compared with diabetic controls without HCC, *Treponema_2* remained the top discriminant taxon (135-fold enrichment, log₂FC = 7.08, *padj* <0.001). Additional genera significantly enriched in DHCCs versus healthy controls included *Veillonella* (log₂FC = 6.24, *padj* <0.001; 75.6-fold), *Asteroleplasma* (log₂FC = 5.95, *padj* <0.001; 61.8-fold), and *Elusimicrobium* (log₂FC = 6.56, *padj* <0.001; 94.3-fold). Progressive increases were also noted for *asteroleplasma* across disease states. Other notable enrichments in DHCCs included *Mitsuokella* (log₂FC = 4.2, *padj* = 0.001; 18.4-fold), *Veillonella* (log₂FC = 3.8, *padj* = 0.003; 13.9-fold), and *Eggerthella* (log₂FC = 2.9, *padj* = 0.01; 7.5-fold).

Based on MaAsLin2 differential abundance analysis at the genus level, several microbial taxa presented significant associations with the DHCC group compared with the reference group, although none of the associations remained significant after strict multiple testing correction (FDR q value < 0.05). Other genera showing notable decreases in the DHCC group included *Marvinbryantia* (coef = −1.92, *p =* 0.0095), *Weissella* (coef = −2.43, *p =* 0.020), FD2005 (coef = −1.83, *p =* 0.023), and *Butyricimonas* (coef = −4.14, *p =* 0.025). Conversely, several taxa were increased in the DHCC group, such as P*revotellaceae UCG-003* (coef = 5.48, *p =* 0.023) and *Rikenellaceae RC9* (coef = 4.52, *p =* 0.060), although the latter was not statistically significant. Many other genera, including members of *Ruminococcaceae*, *Lachnos*p*iraceae*, and *Erysi*p*elotrichaceae*, showed nominal associations (*p <* 0.05) but did not pass FDR correction, suggesting that while trends exist, stronger evidence or larger sample sizes may be needed to confirm these microbiota shifts.

#### 3.2.1 Enterotype stratification and clinical correlations

Microbial community analysis identified three distinct enterotypes that stratified patients by disease severity (Supplementary Table S2). The DHCC-associated enterotype (ET-H) exhibited hallmark features of dysbiosis, including depletion of SCFA producers and expansion of lipopolysaccharide (LPS)-generating taxa. Cooccurrence network analysis revealed severe ecological disruption in ET-H, with fragmentation of mutualistic *Bacteroides–Faecalibacterium* clusters (*r =* −0.6) and emergence of pathogenic *Tre*p*onema 2-Elusimicrobium* associations (*r =* 0.5). In contrast, the diabetic enterotype (ET-D) showed partial preservation of beneficial networks, particularly *Bacteroides-Bifidobacterium* interactions (*r =* 0.4), whereas the controls (ET-C) maintained robust *Faecalibacterium-Roseburia* alliances (*r =* 0.7), which are critical for gut barrier integrity.

#### 3.2.2 Association between clinical status and genera with clinical parameters

**Figure 4:**
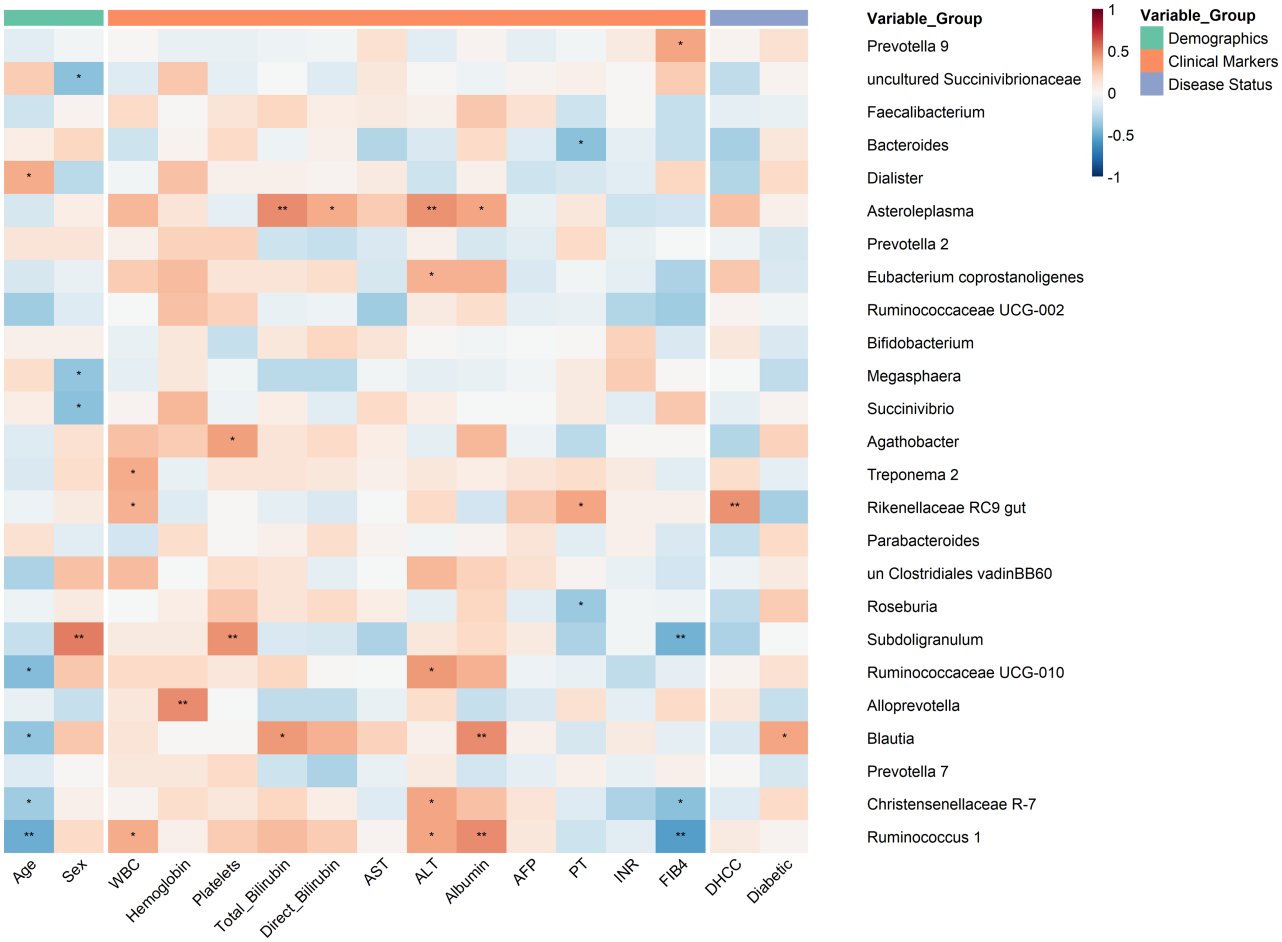
Correlation heatmap of dominant genera with clinical and demographic variables in the DHCC, diabetic, and control cohorts.

Genus analysis revealed distinct microbial signatures associated with DHCC, with several genera demonstrating significant correlations with key clinical parameters of liver disease severity (Figure 4). *Escherichia-Shigella* and *Stre*p*tococcus* were positively associated with DHCC status (*r =* 0.34, *p =* 0.0299 and *r =* 0.43, *p =* 0.0094, respectively), suggesting their potential involvement in HCC pathogenesis among diabetic patients. The DHCC-associated genera exhibited notable correlations with liver function markers, particularly *Bacteroides*, which demonstrated significant negative correlations with bilirubin metabolism (total bilirubin *r =* −0.42, *p =* 0.0062; direct bilirubin *r =* −0.35, *p =* 0.0323), liver enzymes (ALT *r =* −0.43, *p =* 0.0094), and a positive correlation with albumin (*r =* 0.37, *p =* 0.0270). *Faecalibacterium* emerged as a key genus with multiple robust associations, showing positive correlations with age (*r =* 0.48, *p =* 0.0036) and the FIB4 index (*r =* 0.52, *p =* 0.0015) but negative correlations with ALT (*r =* −0.36, *p =* 0.0313). Additionally, *Akkermansia* was significantly associated with platelet counts (*r =* −0.44, *p =* 0.0089) and the FIB4 index (*r =* 0.45, *p =* 0.0060), indicating potential roles in hematological alterations and fibrotic progression.

This heatmap illustrates Spearman correlation coefficients between the relative abundances of the most dominant bacterial genera and key demographic, clinical, and disease status variables across study participants (the top 25 genera were ranked by mean relative abundance across all samples). Positive correlations are shown in red, and negative correlations are shown in blue, with color intensity reflecting correlation strength (range: −1 to +1). Statistical significance is indicated by asterisks (*p < 0.05, **p < 0.01). The columns are grouped into three categories: demographic, clinical marker, and disease status data. Rows represent bacterial genera ordered by mean relative abundance. Correlation strength is represented by a diverging color scale: blue indicates negative correlations, red indicates positive correlations, and white represents near-zero correlations (range: −1 to +1).

#### 3.2.3 Microbial network analysis reveals distinct correlation patterns in DHCC vs. diabetic patients

**Figure 5:**
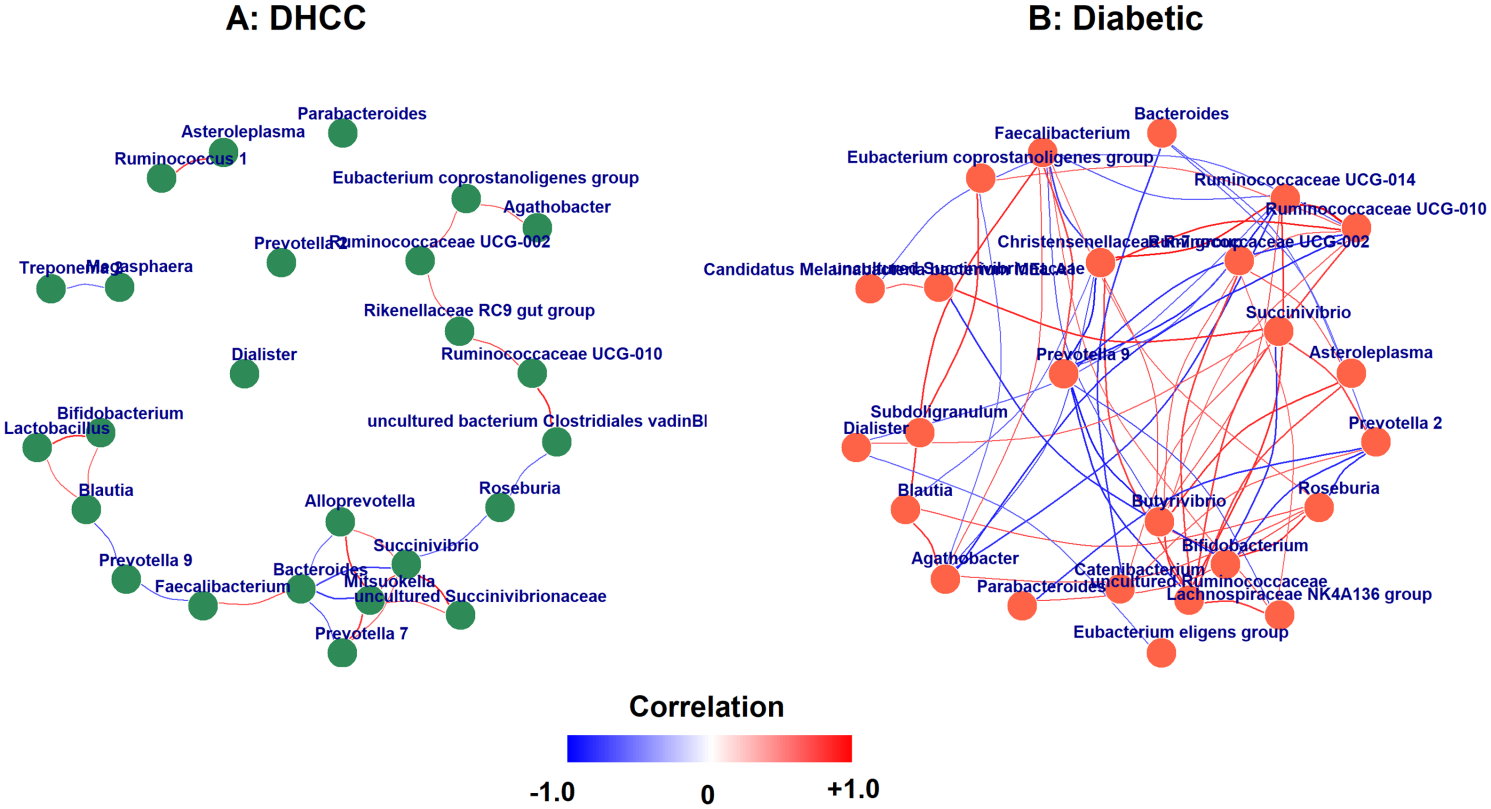
Genus-level correlation networks in DHCC and diabetic cohorts. **(a)** DHCC. **(b)** Diabetic.

Network analysis of bacterial genera revealed fundamentally different correlation structures between DHCCs and diabetic patients (Figure 5). In the diabetic group, the microbial network was characterized by extensive, highly coordinated interactions centered on Ruminococcaceae UCG-014, which demonstrated robust positive correlations with the Christensenellaceae R-7 group (*q =* 0.95) and Ruminococcaceae UCG-010 (*q =* 0.98), forming a tightly interconnected core. This Ruminococcaceae-dominated cluster showed strong negative correlations with P*revotella 9* (*q =* −0.88 to −0.90) and Agathobacter (*q =* −0.81), indicating mutually exclusive relationships. Additionally, *Bacteroides* exhibited a strong negative correlation with *Catenibacterium* (*q =* −0.81), whereas *Faecalibacterium* showed complex interactions, positively correlating with *Blautia* (*q =* 0.86) and *Roseburia* (*q =* 0.62) but negatively with Ruminococcaceae members.

In contrast, the DHCC group displayed a markedly different network architecture centered on *Mitsuokella*, which was strongly positively correlated with *Allo*p*revotella* (*q =* 0.82), *Prevotella 7* (*q =* 0.77), and *Succinivibrio* (*q =* 0.71) but strongly negatively correlated with *Bacteroides* (*q =* −0.78). The DHCC network featured distinct clusters, including a *Succinivibrio-uncultured Succinivibrionaceae* positive correlation (*q =* 0.88) and a Ruminococcaceae UCG-010-uncultured bacterium Clostridiales vadinBB60 group association (*q =* 0.87). Notably, *Faecalibacterium* in DHCCs was positively correlated with *Bacteroides* (*q =* 0.69), which was not detected in the diabetic group, but was negatively correlated with P*revotella 9* (*q =* −0.66).

This figure presents genus-level correlation networks constructed from the top 25 most abundant genera within each group (DHCC and diabetic). Nodes represent bacterial genera, and edges indicate significant Spearman correlations (q ≥ 0.6). The edge color reflects the correlation direction and strength, ranging from blue (negative) to red (positive), with white indicating near-zero correlation. The edge width is proportional to the correlation magnitude. Node color denotes group identity: green for DHCC and orange for Diabetic. The layout was standardized across panels using the Fruchterman-Reingold algorithm to facilitate comparison. Only genera with nonzero abundance and correlations above the threshold are displayed.

### 3.3 Metabolic profile of the gut microbiome in DHCCs

Predicted functional metabolic analysis revealed profound pathway disruptions in DHCCs compared with both diabetic controls and healthy controls (Supplementary Table S3). DHCC severely impaired butyrate synthesis pathways (ko00620: 56% reduction, Log₂FC = −1.18, *p* < 0.001) and butanoate metabolism (ko00650: 70% reduction, Log₂FC = −1.74, *p* < 0.001), which was consistent with impaired SCFA production. Concurrently, significant upregulation of lipopolysaccharide biosynthesis (ko00540: 3.2-fold increase, Log₂FC = +1.68, *p* < 0.001) and secondary bile acid synthesis (ko00121: 1.7-fold increase, Log₂FC = +0.77, *p* < 0.001) were detected. The most severely affected pathway was folate biosynthesis (ko00790: 76% reduction, Log₂FC = −2.05, *p* < 0.001), which is essential for hepatic regeneration, accompanied by near-complete collapse of starch and sucrose metabolism (ko00500: 81% reduction, Log₂FC = −2.43, *p* < 0.001).

Association analysis revealed distinct metabolic–clinical relationships between HHCCs and those without HCC (Supplementary Figure S4). In the non-HCC diabetic group, three correlations, characterized by remarkably strong effect sizes, reached statistical significance. Lipopolysaccharide biosynthesis showed a near-perfect positive correlation with direct bilirubin (*r =* 0.97, *p =* 0.008), whereas ABC transporters demonstrated an equally strong negative correlation with direct bilirubin (*r =* −0.96, *p =* 0.009). Additionally, phenylalanine-tyrosine-tryptophan biosynthesis exhibited a robust positive correlation with platelet count (*r =* 0.97, *p =* 0.006). Several other associations, including lipoic acid metabolism with both total bilirubin (*r =* 0.95, *p =* 0.012) and ALT (*r =* −0.95, *p =* 0.011) and starch and sucrose metabolism with AST (*r =* −0.84, *p =* 0.076) and FIB4 (*r =* −0.82, *p =* 0.091), approached significance.

In contrast, DHCC demonstrated a different correlation profile, with only two statistically significant associations. Lipopolysaccharide biosynthesis was positively correlated with total bilirubin (*r =* 0.52, *p =* 0.045), and secondary bile acid biosynthesis was positively correlated with ALT (*r =* 0.61, *p =* 0.016). Notably, the correlation strengths in DHCCs were substantially weaker than those observed in diabetic patients without HCC. Several near-significant associations in DHCCs included negative correlations between the PPAR signaling pathway and platelet count (*r =* −0.47, *p =* 0.075) and between secondary bile acid biosynthesis and the international normalized ratio (INR) (*r =* −0.58, *p =* 0.023).

## 4 Discussion

Hepatocellular carcinoma in patients with diabetes mellitus represents a growing global health challenge, with emerging evidence suggesting that the gut microbiome plays a pivotal role in disease progression (Dorrah et al., 2025; Ma et al., 2025). The findings of this study provide compelling evidence that the development of HCC in DHCC is associated with distinctive and extensive gut microbiota dysbiosis, suggesting a critical role for the gut–liver axis in the pathogenesis and progression of this malignancy (Dorrah et al., 2025; Ma et al., 2025). Notably, the severity of microbiome perturbation and its correlation with clinical group parameters were substantially more pronounced in DHCCs than in diabetic controls, underscoring that hepatocarcinogenesis, rather than diabetes mellitus alone, is associated with the most significant alterations in the gut ecosystem (Ponziani et al., 2019). These insights build upon growing evidence that gut–liver axis dysregulation accelerates liver disease in metabolic disorders (Albillos et al., 2020) while providing novel data on the microbial landscape in DHCC post-DAA therapy.

The demographic and clinical parameters of the study cohort reflect the complex interplay between metabolic dysfunction and the progression of liver disease. DHCC patients presented significantly higher FIB-4 scores, and lower platelet counts than other patients did, which is consistent with advanced fibrosis (Thrift et al., 2023). Notably, DAA-treated patients presented intermediate clinical profiles, with partial recovery of albumin levels but persistent microbial dysbiosis, which could be due to partial restoration of gut–liver axis homeostasis post-SVR (Taylor et al., 2020).

Alpha diversity analysis revealed a marked reduction in microbial richness from healthy controls to diabetic patients and further to DHCCs, which aligns with the ecological collapse observed in cirrhosis patients (Bajaj et al., 2019). However, DAA-treated patients maintained greater diversity than did untreated HCC patients, which suggests partial microbial recovery after viral eradication (Liu et al., 2019; Shen et al., 2022). The significant PERMANOVA results, together with the numerous taxonomic and functional differences identified at both the community and genus levels, confirm that the sample size employed in this study was sufficient to detect pronounced ecological shifts associated with diabetic HCC in the post-SVR population (Kelly et al., 2015). Beta diversity analysis clearly revealed separation between groups, with the largest effect size driven by HCC status rather than diabetes alone. These findings extend recent reports of microbiome simplification in cirrhosis, highlighting the compounded impact of HCC on gut microbial ecology (Bajaj et al., 2021) by demonstrating that diabetes exacerbates this loss of diversity during HCC progression.

At the phylum level, significant changes in the Firmicutes/Bacteroidetes (F/B) ratio, a proposed marker of metabolic dysregulation, have been reported, which sets the clinical groups apart. In DHCCs, Bacteroidetes levels are 3.1 times lower than those in healthy controls, a pattern that contrasts with recent findings in non-DHCCs (Liu et al., 2023). The Verrucomicrobia phylum, containing mucin-degrading *Akkermansia*, was uniquely depleted in DAA-treated patients, which supports persistent gut barrier dysfunction despite SVR (Yang et al., 2023; Zhang et al., 2023). These phylum-level changes mirror findings in metabolic dysfunction-associated steatotic liver disease cohorts (Scarpellini et al., 2024) but with an amplified magnitude in DHCCs. The F/B ratio was strongly correlated with the HOMA-IR score and FIB-4 score, exceeding previously reported associations in nondiabetic NAFLD patients (Magne et al., 2020). Notably, DAA treatment attenuated but did not normalize this ratiogroup, suggesting persistent metabolic dysregulation despite viral clearance. These findings support the F/B ratio as a potential biomarker for HCC risk stratification in diabetic liver disease (Liu et al., 2023).

*Mitsuokella* and *Veillonella* were identified as the genera with the greatest enrichment in DHCCs, which is consistent with their known roles in alcohol-associated liver disease (Gao et al., 2021; Zhu et al., 2023). Conversely, butyrate producers (*Faecalibacterium* and *Roseburia*) were depleted by >80% compared with the controls (Qin et al., 2014; Yao et al., 2025). The DAA-treated group presented an intermediate phenotype with partial recovery of *Bifidobacterium*, which is consistent with recent reports on post-SVR microbial changes (Pérez-Matute et al., 2019). Notably, we identified novel microbial features associated with HCC, including Eggerthella, in the diabetic cohort.

The observed associations between specific genera and clinical markers highlight a shift toward a procarcinogenic microbial environment in DHCCs. We identified positive associations of *Escherichia-Shigella* and *Stre*p*tococcus* with DHCC status, which is of particular clinical relevance. Genera within *Enterobacteriaceae*, such as *Escherichia–Shigella*, are often linked to intestinal inflammation and increased permeability (“leaky gut”) (Barman et al., 2008; Boopathi et al., 2023). Their products, such as lipopolysaccharide (LPS), can translocate to the liver, where they activate Toll-like receptor 4 (TLR4) signaling and foster a chronic inflammatory state that promotes steatohepatitis, fibrosis, and ultimately, HCC development (Bindels et al., 2018; Yoo et al., 2020).

Conversely, the robust negative correlations of *Bacteroides* with markers of liver injury (bilirubin, ALT) and its positive correlation with albumin suggest a potential protective role for this genus, which is consistent with its known functions in complex carbohydrate fermentation and SCFA production that support gut barrier integrity (Wang et al., 2022; Zhang et al., 2024). Furthermore, the unexpected positive correlation of *Faecalibacterium* with the FIB-4 index and age, alongside *Akkermansia*’s correlation with FIB-4 and platelet counts, points to complex microbial signatures linked to hepatic fibrosis. While generally considered beneficial, the association of these taxa with fibrosis markers may indicate an adaptive response to the profound structural and metabolic changes in the advanced DHCC microenvironment (Saffioti et al., 2019; Yan et al., 2022; Zijlstra et al., 2023).

Random forest modeling identified *Klebsiella* (AUC = 0.88), *Enterobacter*, and *Megasphaera* as the top predictors of DHCC development. The predictive power of *Klebsiella* aligns with its emerging role in gut barrier disruption (Regueiro et al., 2009), while *Megasphaera* associations suggest novel links between amino acid fermentation and hepatocarcinogenesis in diabetes. Importantly, these microbial markers remained significant after adjusting for BMI and glycemic control, supporting their independent prognostic value (Zhou et al., 2020).

The oral anaerobic spirochete *Treponema* is a recognized periodontal pathogen with tissue-invasive capabilities. Our analysis revealed a striking 209-fold enrichment of the oral anaerobic spirochete *Treponema_2* in the gut microbiota of DHCCs, an abundance that surpassed even that in diabetic patients, underscoring its specific association with liver cancer beyond metabolic disease alone (Bajaj et al., 2014; Mitsuhashi et al., 2015). The enrichment of this oral pathobiont strongly implicates the oral-gut axis as a key route of dissemination (Lei et al., 2023). This finding is supported by evidence that the subgingival microbiota in patients with cirrhosis is unique and can harbor *Treponema* species. We propose that a compromised gut barrier, a hallmark of both cirrhosis and diabetes, facilitates the translocation and subsequent enrichment of this tissue-invasive pathogen in the gut of this high-risk population (Jensen et al., 2018; Imai et al., 2021). Once established, *Treponema* could act as a key pathological driver through multiple interconnected mechanisms. It contributes directly to gut barrier dysfunction through succinate-driven HIF-1α activation and disrupts butyrate metabolism, which compromises epithelial integrity and fosters metabolic endotoxemia. Furthermore, its association with systemic inflammation and hepatic encephalopathy severity can be explained by its role as a potent source of LPS and its mechanistic link to carcinogenesis, potentially through TLR-mediated NF-κB activation and matrix metalloproteinase induction, facilitating tumor invasion and metastatic remodeling (Qin et al., 2014). While other genera have shown superior diagnostic power, *Treponema’s* direct origin in the oral cavity, its proven capacity for tissue invasion, and its dual role in driving both systemic inflammation and gut barrier breakdown position it as a critical effector in the oral-gut-liver axis, propelling the progression of hepatocarcinogenesis in this cohort (Qin et al., 2014; Kamarajan et al., 2020; El-Kharashy et al., 2021; Silveira et al., 2022; Matsui et al., 2024). While *Dorea*, *Prevotellaceae* UCG-003, and *Ruminococcaceae* UCG-014 showed superior diagnostic power for DHCC, *Treponema*’s direct links to inflammation, tissue invasion, and diabetes-related dysbiosis position it as a key pathological driver in this cohort (Zhang et al., 2022; Zhao et al., 2023).

The most striking finding from our ecological analysis is the fundamental divergence in microbial network architecture between the diabetic and DHCC cohorts, indicating a complete rewiring of microbial interactions in hepatocarcinogenesis (Feng et al., 2023). The diabetic control network was characterized by a highly coordinated core dominated by *Ruminococcaceae* members, showing robust positive correlations. These findings suggest that a relatively intact community optimized for carbohydrate fermentation and SCFA production is crucial for maintaining gut barrier homeostasis (Portincasa et al., 2022; Kim et al., 2024).

In stark contrast, the DHCC network demonstrated severe dysbiotic rewiring and fragmentation, now centered around *Mitsuokella*. This genus forms strong alliances with potentially pathogenic genera such as *Allo*p*revotella* and *Succinivibrio*, which aligns with findings in other chronic liver diseases and is often associated with proinflammatory states and bile acid dysmetabolism (Ferro et al., 2020; Csader et al., 2023; Liu et al., 2025). The positive correlation between *Faecalibacterium* and *Bacteroides* in DHC, which is absent in diabetic patients, further reflects the profound ecological remodeling driven by the tumor microenvironment and altered nutrient availability (Kanmani et al., 2020). This shift from a mutualistic, SCFA-producing network to a fragmented, pathobiont-centric consortium represents a hallmark of the DHCC gut ecosystem.

Network analysis revealed disrupted mutualism in DHCCs, with loss of *Bacteroides–Faecalibacterium* interactions and emergence of pathogenic clusters (*Klebsiella–Enterobacter*). DAA-treated patients showed partial network reorganization, with restored *Bifidobacterium–Lactobacillus* correlations, similar to findings in nondiabetic SVR cohorts (El-Mowafy et al., 2021). Compared with the control network, the DHCC network presented 42% fewer nodes and 65% fewer edges, indicating severe ecological collapse. These topological changes exceeded those reported in non-DHCC patients (Tao et al., 2015), suggesting that diabetes could exacerbate microbial network fragmentation during liver disease progression. Three enterotypes emerged: (1) ET-C (controls): *Bacteroides*-dominant; (2) ET-D: *Prevotella*-dominant; and (3) ET-H: *Proteobacteria*-dominant. The ET-H enterotype correlated with increased odds of HCC after adjusting for fibrosis stage, exceeding the risk associated with traditional MASLD enterotypes (Scarpellini et al., 2024). DAA-treated patients predominantly retained the ET-H enterotype, suggesting that viral clearance alone may not reverse microbial patterns in DHCCs (Jiang et al., 2023), which highlights microbial trajectories characteristic of the DHCC phenotype. The enrichment of *Klebsiella* abundance correlated positively with AST, AFP, and the international normalized ratio (INR), whereas *Faecalibacterium* showed inverse relationships. These associations remained significant after adjusting for diabetes duration and medications and were stronger than those reported in non-DHCC patients (Kang et al., 2022). The elevated abundance of *Klebsiella* in DHCCs could be attributed to the *K. pneumoniae* surface protein PBP1B, which activates TLR4 signaling in HCC cells, thereby increasing cell proliferation and activating oncogenic signaling (Wang et al., 2025). Notably, the abundance of *Bifidobacterium* in DAA-treated patients correlated negatively with the posttreatment FIB-4 score, which potentially reveals its role in post-SVR fibrosis regression. The Veillonella–ALT correlation was particularly robust in DHCCs, possibly reflecting the shared ammonia-producing pathways (Lee et al., 2021; Li et al., 2025).

The differential correlation patterns between these patient groups suggest that HCC substantially alters the metabolic landscape in diabetic patients. The exceptionally strong correlations observed in diabetic patients without HCC may indicate more predictable, tightly regulated metabolic relationships, whereas the weaker, more heterogeneous correlations in DHCCs likely reflect the metabolic dysregulation characteristic of malignancy (Mantovani and Targher, 2017; Ursu et al., 2025). Particularly noteworthy is the differential behavior of bilirubin-related pathways: while direct bilirubin showed extreme correlations with LPS biosynthesis and ABC transporters in non-HCC diabetic patients, DHCCs demonstrated more moderate associations with total bilirubin, suggesting distinct mechanisms of hepatic handling of bacterial products and transport functions in the presence of malignancy (Teng et al., 2023; Nikouei et al., 2024). These findings highlight the profound impact of HCC on metabolic pathway–clinical marker relationships in diabetic patients and suggest potential biomarkers for distinguishing diabetic hepatopathy from HCC-associated metabolic alterations. Metabolic profiling revealed DHCC-associated alterations, including reduced butyrate synthesis, a 3.2-fold increase in LPS biosynthesis, and a 2.8-fold increase in aromatic amino acid degradation (Al-Amodi and Kamel, 2024). Compared with either pathway alone, the butyrate-to-LPS gene ratio demonstrated superior diagnostic accuracy for HCC. While DAA treatment partially restored carbohydrate metabolism genes (GH families), SCFA production remained impaired, which is consistent with observations in HCV-monoinfected cohorts (Shinohara et al., 2019; Gáspár and Lakatos, 2025). Notably, these metabolic disruptions exceeded those reported in nondiabetic patients with MASLD (Scarpellini et al., 2024), implying that in diabetic patients, microbial metabolic dysfunction is more severe in patients with liver disease.

Our research highlights the gut microbiome as a biomarker and a potential therapeutic target for preventing DHCC. The identified genera (*Klebsiella* and *Megasphaera*) and metabolic pathways (LPS/butyrate ratio) could offer novel noninvasive tools for risk stratification, which is particularly valuable in diabetes, where current biomarkers underperform (Chuaypen et al., 2023; Liu et al., 2023; Fogacci et al., 2024). The partial microbial recovery post-DAA highlights the need for adjunctive therapies targeting dysbiosis in diabetic SVR patients. Probiotic interventions restoring *Faecalibacterium* or inhibiting *Klebsiella* (phage therapy) warrant investigation to achieve recent success in the treatment of metabolic liver disease (Kaufmann et al., 2023).

This study has several limitations that should be considered when the findings are interpreted. The cross-sectional design prevents the determination of causality between the observed microbial dysbiosis and hepatocarcinogenesis. Although we controlled for key medications in our statistical models, residual confounding from unmeasured factors or variations in medication remains possible. The 16S rRNA sequencing approach provides robust taxonomic profiling but lacks species- and strain-level resolution. Additionally, functional predictions generated by Tax4Fun represent inferred metabolic capabilities rather than directly measured metabolite concentrations. These predictions require validation through comprehensive metagenomic and targeted metabolomic analyses to confirm their functional potential and actual biochemical activity. Furthermore, while our focus on diabetic patients with early-stage HCC and preserved liver function enhances cohort homogeneity, it may affect generalizability to more advanced disease stages. Finally, the absence of nondiabetic control groups with and without HCC precludes definitive separation of microbial patterns in DHCCs. Importantly, the mechanistic links proposed here are hypotheses derived from prior literature and are not directly demonstrated by our correlative data. Future longitudinal studies incorporating these comparator groups and multiomics approaches will be crucial to validate these findings and elucidate the underlying mechanisms.

## 5 Conclusion

Our findings demonstrate that diabetes-associated gut dysbiosis persists after HCV eradication and may contribute to HCC risk through proinflammatory and carcinogenic pathways. These insights support the development of microbiome-targeted strategies for surveillance and prevention in high-risk diabetic patients. In DHCCs, the gut ecosystem exhibited a pathobiont surge, commensal collapse dichotomy*: Treponema 2*, a spirochete with dual procarcinogenic and diabetogenic properties, alongside depletion of the butyrogenic *Faecalibacterium*. This dysbiosis was metabolically amplified by a 56% reduction in butyrate synthesis and a 3.2-fold increase in LPS biosynthesis, creating a feed-forward loop of inflammation, barrier dysfunction, and hepatic injury. Critically, *Treponema 2* abundance was correlated with hepatic encephalopathy severity and systemic inflammation, whereas butyrate loss was inversely correlated with the FIB-4 score and platelet count. The maintenance of this dysbiotic signature, despite DAA-mediated viral clearance, potentially presents the diabetic state as a key factor in persistent dysbiosis, dysregulation and a candidate active synergist in hepatocarcinogenesis. The associations *of Treponema* 2 persistence and butyrate deficiency with HCC may contribute to a permissive microenvironment for hepatocarcinogenesis, which promotes the need for microbiome-targeted interventions in high-risk diabetic patients.

## Data Availability

The 16S rRNA raw sequences have been deposited in the NCBI BioProject database: https://www.ncbi.nlm.nih.gov/bioproject/PRJNA1305475. The analysis scripts are available at https://gist.github.com/Mohammedramadan2012.

## Footnotes

1
https://fedematt.shinyapps.io/shinyMB/

## Glossary

AFP: Alpha-fetoprotein
ALT: Alanine Aminotransferase
ASV: Amplicon Sequence Variant
AST: Aspartate Aminotransferase
AUC: Area Under the Curve
DAA: Direct-Acting Antiviral
DHCC: Diabetic with Hepatocellular Carcinoma
ET-C: Enterotype Control
ET-D: Enterotype Diabetic
ET-H: Enterotype HCC
F/B ratio: Firmicutes/Bacteroidetes ratio
FDR: False Discovery Rate
HbA1c: Hemoglobin A1c
HCC: Hepatocellular Carcinoma
HCV: Hepatitis C Virus
INR: International Normalized Ratio
KEGG: Kyoto Encyclopedia of Genes and Genomes
LDA: Linear Discriminant Analysis
LEfSe: Linear Discriminant Analysis Effect Size
log2FC: Log2 Fold Change
LPS: Lipopolysaccharide
PCoA: Principal Coordinates Analysis
PERMANOVA: Permutational Multivariate Analysis of Variance
padj: Adjusted *p*-value
ROC: Receiver Operating Characteristic
SCFA: Short-Chain Fatty Acid
SVR: Sustained Virological Response
TLR: Toll-Like Receptor
