# Supplementary material for: The gut microbiome in diabetic patients with hepatocellular carcinoma: distinct bacterial compositional shifts after hepatitis C virus eradication
Source: Front Microbiol. 2026 Jan 9;16:1693345. doi: 10.3389/fmicb.2025.1693345 (PMC12827569; doi:10.3389/fmicb.2025.1693345)
Supplement: Supplementary file 2 [file Supplementary_file_2.docx]

**The Gut Microbiome in Diabetic Hepatocellular Carcinoma: Distinct Bacterial Compositional Shifts after Hepatitis C Virus Eradication**

**Table S1. Comprehensive Model Validation Metrics**

| Validation Approach | Metric | Value | Description |
| --- | --- | --- | --- |
| **Cross-validation** | Iterations | 50 | 10 repeats of 5-fold CV |
|  | Mean AUC | 0.739 | Primary performance metric |
|  | AUC SD | 0.062 | Performance stability |
| **Hold-out Test** | AUC | 0.537 | Independent validation |
| **Permutation Test** | Iterations | 1000 | Statistical significance testing |
|  | True Model AUC | 0.778 | Unpermuted performance |
|  | p-value | 0.024 | Statistical significance |
| **Model Parameters** | Trees | 500 | Random Forest setting |
|  | Split Criterion | Gini impurity | Feature selection method |
|  | Random Seed | 123 | Reproducibility setting |

*Note: This table provides comprehensive technical details of the Random Forest classification analysis.

**Table S2. Microbial Enterotypes in DHCC Progression**

| **Enterotype** | **Key Genera (Mean Relative Abundance)** | **Associated Group** | **Clinical Features** |
| --- | --- | --- | --- |
| **ET-H (DHCC)** | 1. *Treponema 2* (5.74%) | DHCC | Elevated AST/ALT, thrombocytopenia |
|  | 2. *Asteroleplasma* (1.29%) |  | (Platelets: 165.8 ± 66.3 ×10^3^/L) |
|  | 3. *Elusimicrobium* (0.05%) |  | Hyperammonemia, INR elevation |
|  | 4. *Veillonella* (0.03%) |  | (INR: 1.18 ± 0.2) |
|  | 5. *Ruminococcus gnavus group* (0.04%) |  |  |
| **ET-D (Diabetic)** | 1. *Prevotella 9* (20.7%) | Diabetic | Intermediate metabolic dysregulation |
|  | 2. *Bacteroides* (12.9%) |  | HbA1c: 7.8 ± 1.2 |
|  | 3. *Bifidobacterium* (3.7%) |  | Mild hepatic steatosis |
|  | 4. *Parabacteroides* (1.9%) |  |  |
|  | 5. Ruminococcaceae UCG-014 (1.5%) |  |  |
| **ET-C (Control)** | 1. *Faecalibacterium* (9.99%) | Control | Normal liver function tests |
|  | 2. *Bacteroides* (9.57%) |  | Platelets: 250 ± 25 ×10^3^/L |
|  | 3. *Roseburia* (6.9%) |  | FIB-4: 1.29 ± 0.3 |
|  | 4. *Agathobacter* (4.0%) |  |  |
|  | 5. *Subdoligranulum* (1.5%) |  |  |

- OR (ET-H vs ET-C): 4.5 (95% CI: 2.1–9.8), derived from univariable logistic regression.

**Figure S1.** Rarefaction Curves Depicting Sequencing Depth and Observed ASVs Across Study Groups.

**
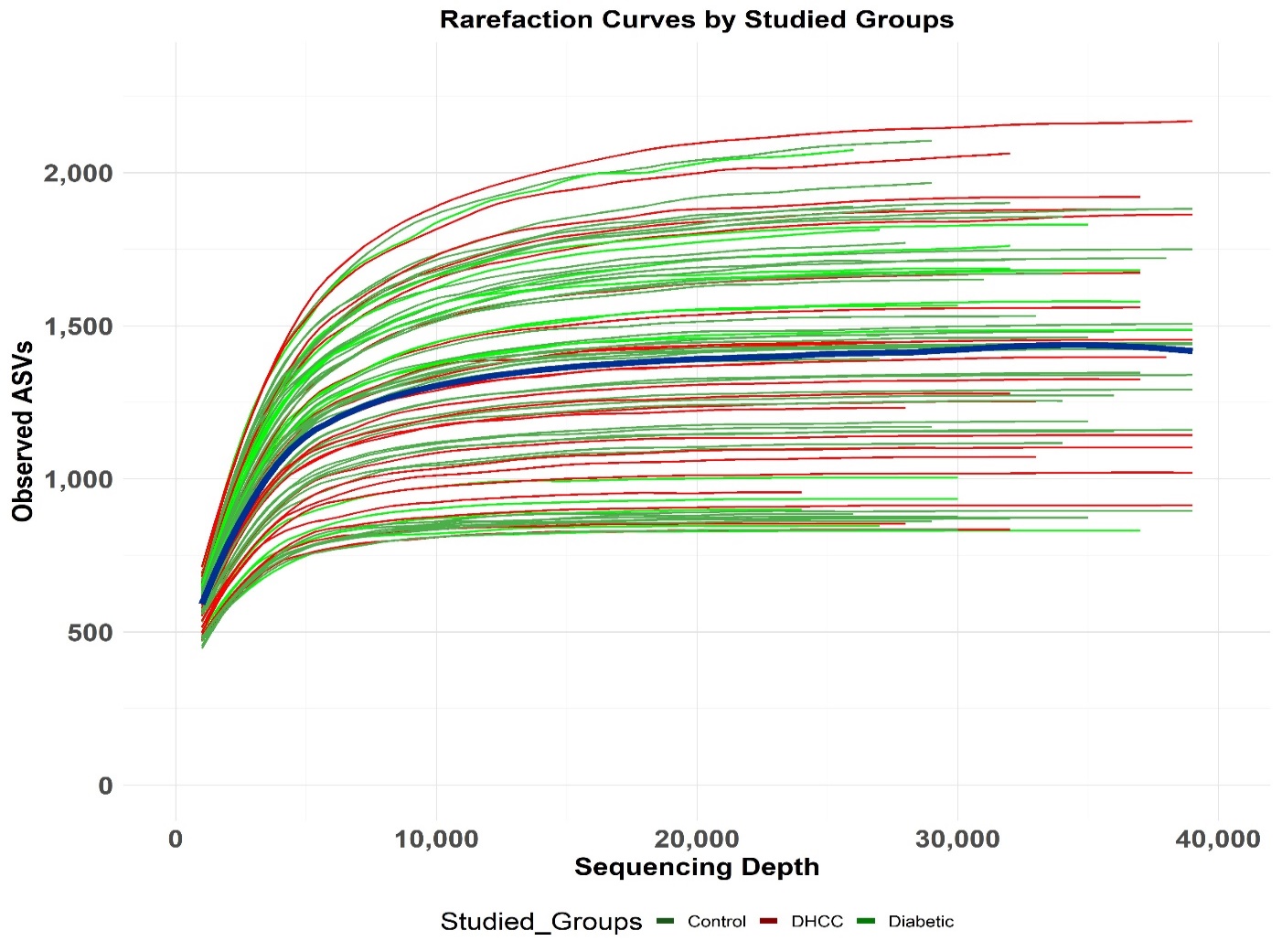
**

**Figure S1.** Rarefaction Curves Depicting Sequencing Depth and Observed ASVs Across Study Groups.

Rarefaction curves illustrate alpha diversity saturation for study cohorts: healthy controls (green), diabetic patients without HCC (blue), and diabetic patients with hepatocellular carcinoma (DHCC, red). The x-axis represents sequencing depth (number of reads), while the y-axis shows the number of observed amplicon sequence variants (ASVs). Curves demonstrate that sequencing depth was sufficient to capture the majority of microbial diversity, with DHCC samples exhibiting lower richness compared to controls, indicating pronounced dysbiosis.

**Gut Microbiome Signature Distinguishes Diabetic Patients with HCC**

We investigated whether the gut microbiome could distinguish diabetic patients with hepatocellular carcinoma (DHCC) from those without HCC (Diabetic). Using a Random Forest classifier, we identified a microbial signature with significant discriminatory power.

The model demonstrated robust performance across multiple validation approaches. It achieved a mean cross-validation AUC of 0.739 (95% CI: 0.722-0.756), indicating consistent generalizability across data subsets (Figure 1C). Permutation testing confirmed the model's performance was significantly better than chance, with the true model (AUC = 0.778) exceeding permutation-based AUC values (p = 0.024) (Figure 1D).

Feature importance analysis with FDR correction revealed specific bacterial genera driving classification accuracy (Figure 1A). Several genera from the *Enterobacteriaceae* family, including *Klebsiella* (0.88) and *Enterobacter* (0.85) featured prominently among the top predictors.


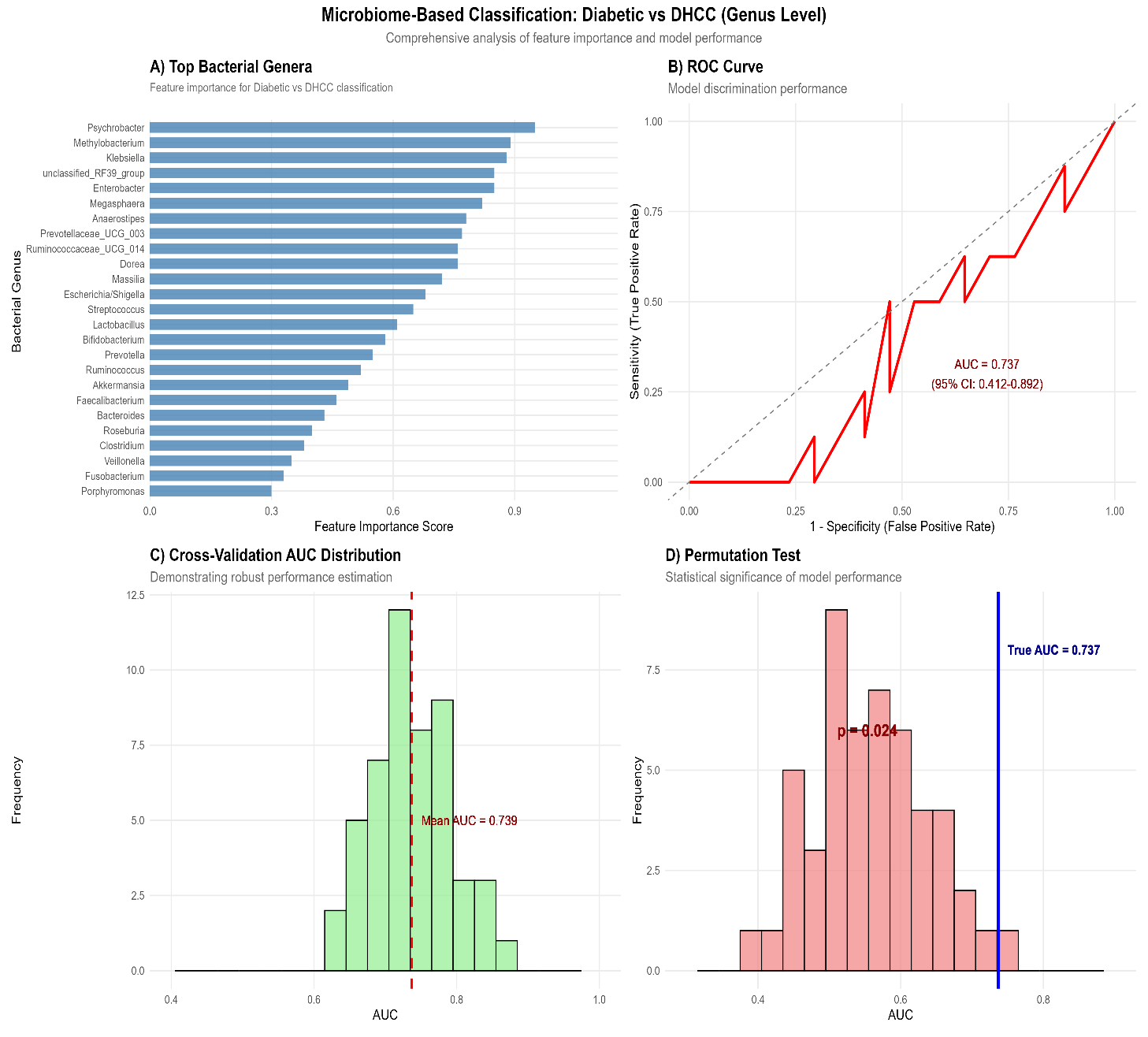


**Figure S2**: Comprehensive validation figure including (A) ROC curve with DeLong's 95% CI, (B) Precision-Recall curve, (C) Cross-validation AUC distribution, and (D) Permutation test results with 1000 iterations. Includes ROC curve (AUC with 95% CI), Precision-Recall curve (PR-AUC), cross-validation AUC distribution, and permutation test results. Confusion matrices are provided in Supplementary Table S1.

**Figure S3.** ROC Curves for Top Discriminative Bacterial Genera Across DHCC and diabetics.


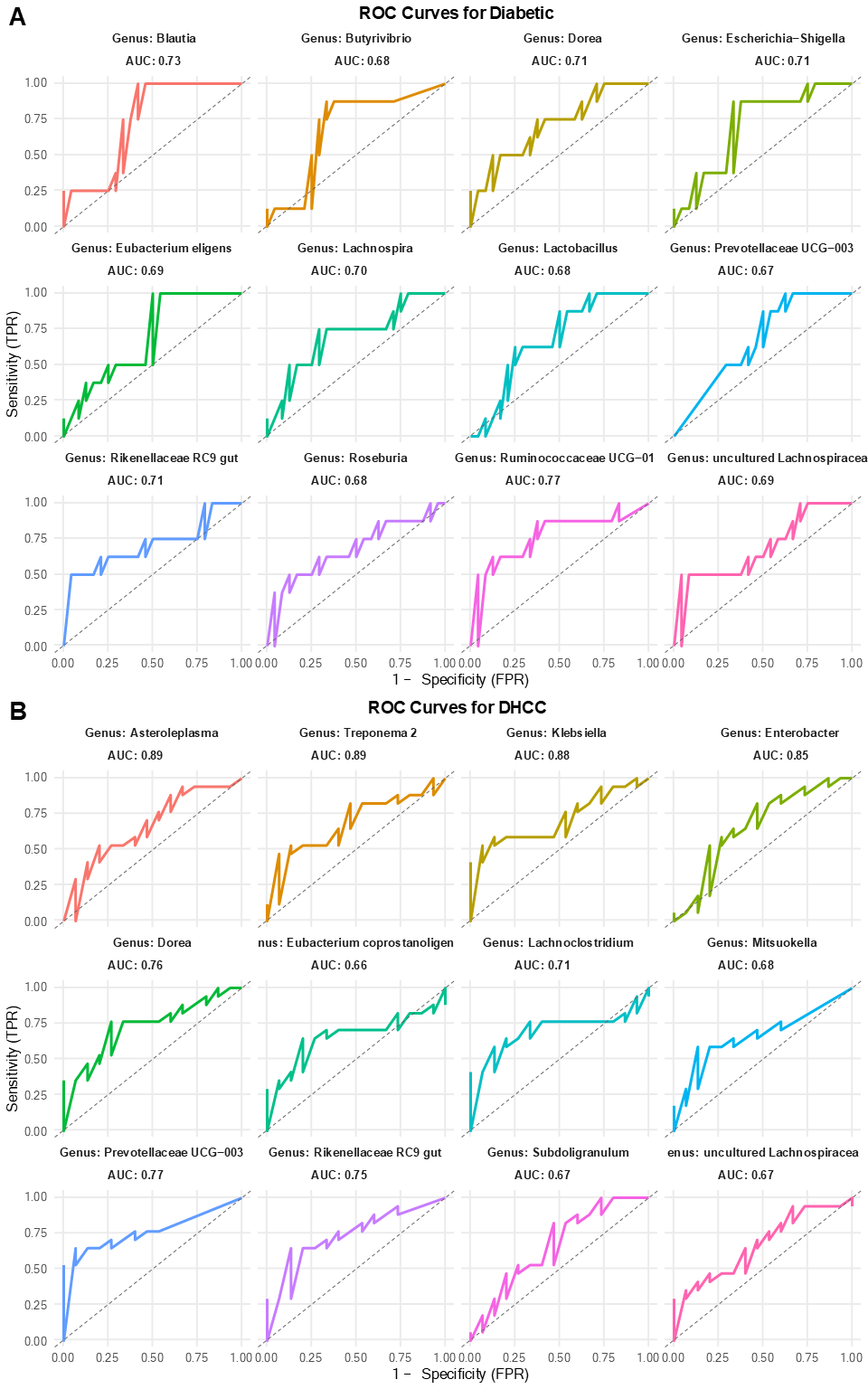


**Figure S3.** Receiver Operating Characteristic (ROC) curves for the most discriminative bacterial genera identified in three clinical groups: Diabetic (A) and DHCC (B) Each subplot illustrates the diagnostic performance of a specific genus, with sensitivity plotted against 1-specificity. The Area Under the Curve (AUC) values are provided to quantify the classification accuracy of each genus in distinguishing between groups.

**Figure S4: Metabolic Pathway Correlations with Clinical Markers Across Diabetic Liver Disease Spectrum**


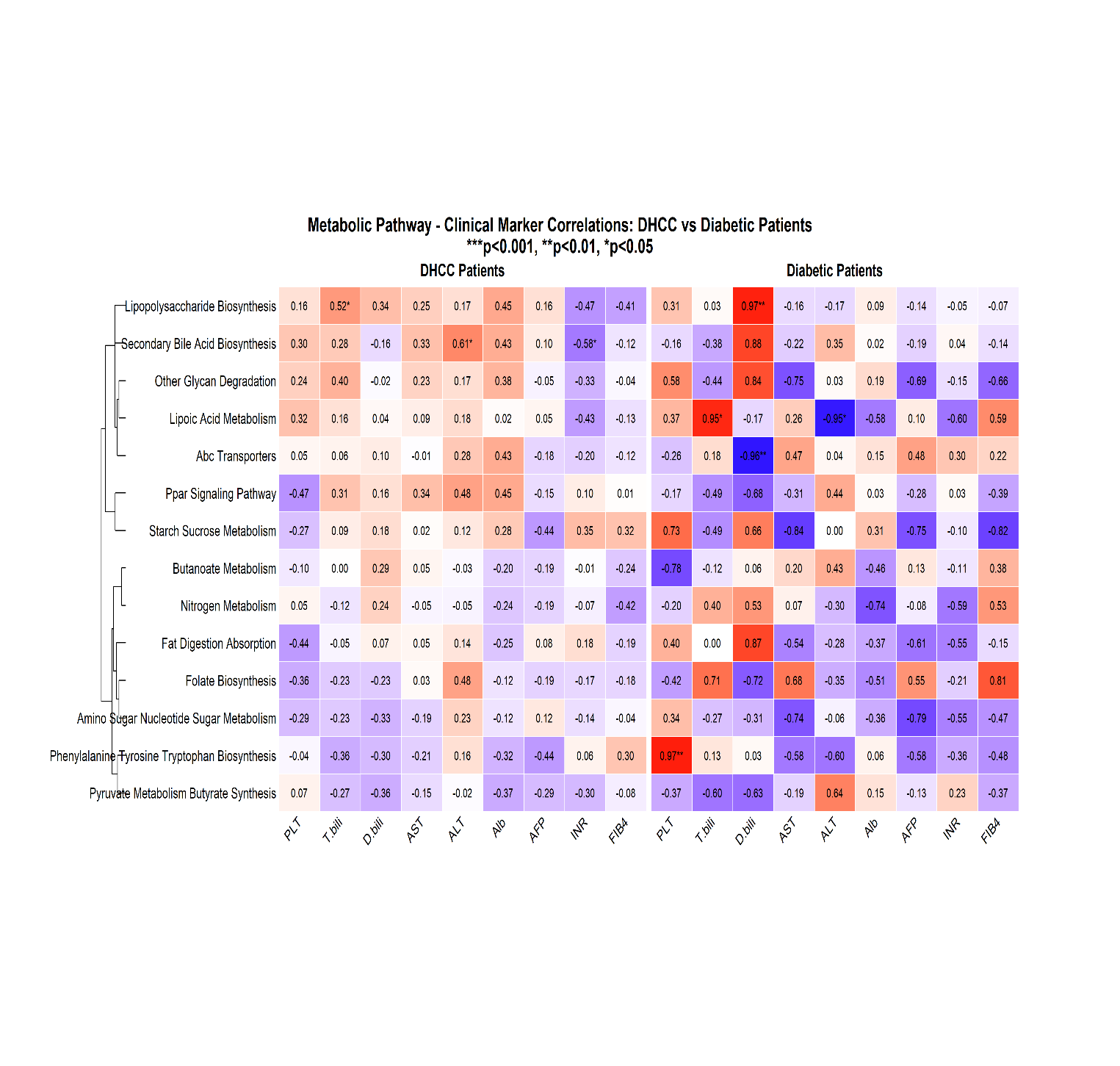


**Figure S4: Metabolic Pathway Correlations with Clinical Markers Across Diabetic Liver Disease Spectrum**

Heatmap visualization of Spearman correlation coefficients between metabolic pathways and clinical markers in diabetic patients without hepatocellular carcinoma (left) and diabetic patients with HCC (right). Statistically significant correlations (p<0.05) are indicated with asterisks: *p<0.05, **p<0.01, ***p<0.001. The color scale represents correlation strength from -1 (blue, negative correlation) to +1 (red, positive correlation).
